# Psychological Well-Being of Female-Headed Households Based on Age Stratification: A Nationwide Cross-Sectional Study in South Korea

**DOI:** 10.3390/ijerph17186445

**Published:** 2020-09-04

**Authors:** Min Kwon, HyungSeon Kim

**Affiliations:** 1 Department of Nursing, The University of Suwon, 17 Bondam-eup, Hwaseong-si 18323, Gyeonggi-do, Korea; mink@suwon.ac.kr; 2 Department of Nursing, Bucheon University, 56 Sosa-ro, Bucheon-si 14774, Gyeonggi-do, Korea

**Keywords:** female-headed households, WHO, well-being, health equity

## Abstract

The female-headed household is a new vulnerable group associated with health inequality. The purpose of this study was to analyze psychological well-being and related factors among Korean female-headed households based on age stratification. This was a secondary analysis of data extracted from the fifth Korean Working Conditions Survey (2017), which included a total of 9084 female-headed households. Their psychological well-being was measured by the WHO-5 well-being index. A total of 39.8% of female-headed household workers were psychologically unhealthy. Among them, 2.2% of those aged 15–30 years old, 8.1% aged 30–50 years old, and 29.5% over aged 50 years old were unhealthy. In the age group of 15–30 years old, depression/anxiety was negatively associated with psychological well-being. In the age group of 30–50 years old, living alone, musculoskeletal pain, fatigue, and depression/anxiety were negatively associated with psychological well-being. In the age group over 50 years old, low education level, living alone, low income, musculoskeletal pain, fatigue, and depression/anxiety were negatively associated with psychological well-being. The psychological well-being perceived by female households is complex and goes beyond economic poverty and dependent burdens. Therefore, a multidimensional support strategy should be included in the concept of social deprivation, and a preventive approach is needed to establish a support system.

## 1. Introduction

Health equity aims to continuously improve health levels of socially vulnerable groups, which is in line with the ultimate goal set by the World Health Organization (WHO). In ‘Health for All (HFA)’, health promotion includes fundamental principles addressing inequality and ensuring health equity [1]. Health inequality is generally affected by sociocultural characteristics, lifestyles, and standards of living. Attention is focused on female-headed households as a new vulnerable group that is occurring globally [2].

In the past, research on health inequality was conducted mainly on social status according to gender, occupation, income, and educational background [3]. Recently, the diversity of family types has indicated that female-headed households are another poor group. The female-headed household is where a woman is in charge of the family livelihood, a comprehensive concept that can include the absence of a spouse, the presence of a spouse, or a single woman. This could include a female-headed household without a spouse due to bereavement or divorce, a woman who has a husband who cannot take responsibility as a family provider, an unmarried woman who is responsible for the livelihood of her family, or a woman who lives alone. Weaknesses in female-headed households should be considered with gender-related disadvantages related to quality such as working conditions and job security, and economic activities related to family livelihoods. Related to socio-economic characteristics, women experience social problems due to gender discrimination in the labor market. In addition, women are affected by lifecycle roles during pregnancy, childbirth, and parenting.

Many underlying factors have contributed to the increase in female-headed households: family breakup is closely related to increased divorce rates, the rapid increase in single-woman households, and women’s relatively long life expectancies [4]. The proportion of female-headed households has increased continuously to 31.2%; 12.7% higher than it was in 2019. Additionally, the number of single-woman households increased 2.2 times compared to 2000 [5]. The proportion of female heads of house is expected to continue increasing as the number of single and divorced household increases. This is a global trend and social support issues and health management solutions are discussed along with poverty problems [6]. In terms of social support in particular, it has been reported through systematic reviews that mental health support is very effective [7]. Emotional and instrumental support for female-headed households, rather than simply supporting poverty, a problem that female householders are facing, has been confirmed to prevent additional health problems. Therefore, it is necessary to identify the degree of psychological health female householders perceive.

Most previous studies [8,9,10,11,12] on female-headed households have focused on cases where women inevitably become head of households in situations of war or in crisis situations in socioeconomically vulnerable countries. However, as South Korea is a member of the Organization for Economic Cooperation and Development (OECD), the vulnerability experienced by female-headed households in Korea means that it is different from these countries. In Korea, women are responsible for most of the housework. Women who are more socioeconomically vulnerable are more likely to be unhealthy due to the burden of their work as a living as well as their role in family care including housework [13].

The gender roles given to women in each developmental life cycle varies [14]. In general, women’s social participation declines around 30 years of age, due to heightened demands from her family. After that, it temporarily increases after the growth of her children, but middle-aged women are once again restricted in social participation due to stable employment and health difficulties [15]. Numerous studies [16,17,18,19,20] have been conducted to identify the associated factors on psychological well-being among women; however, studies based on age stratification are few, although the gender roles given to women in each developmental life cycle varies. Therefore, this study aimed to analyze the psychological well-being and related factors among Korean female-headed households with age stratification, using a nationally representative Korean sample from the fifth Korean Working Conditions Survey (KWCS, 2017).

## 2. Materials and Methods

### 2.1. Study Design and Data Collection

This was a secondary data analysis using the fifth KWCS as a cross-sectional study to identify the psychological well-being of female-headed households and the associated factors by age group. The KWCS targets wage workers (15 years or older) in South Korea and aims to understand overall working environment such as working type, employment type, occupation, type of industry, risk exposure, employment stability, etc. The survey was conducted with a surveyor who visited a household within the survey area, explained the questionnaire and the confidentiality of respondents, and interviewed one wage worker aged 15 or older in the household after obtaining consent. In this study, 9084 female-headed household workers from a total of 50,493 wage workers were included after excluding those with missing data or those who failed or refused to respond.

### 2.2. Variables

Regarding the study variables, demographic characteristics and health related characteristics were selected among variables obtained from the KWCS data by referring to items reported as factors related to psychological well-being in previous studies [3,7,21,22,23].

#### 2.2.1. Demographic Characteristics

The following covariates related to socio-economic and working environment structural factors were considered: age, education level, household type, monthly income, occupation type, employment status, shift work, and night shift. Age group of female heads of house was divided into three categories: 15–29, 30–49, and over 50 years old. Education level was divided into three categories: middle school or less, high school, college or more. Household type was divided into six categories: single household living alone, living with a spouse, living with children, living with a spouse and children, living with parents, and living with other family members or relatives. Monthly income was divided into four categories: less than one million Korean Won (KRW), more than one million and less than two million KRW (1–1.99), more than two million and less than three million KRW (2–2.99), and more than three million KRW. Job type was divided into four categories: administrator or expert, office worker, sales and service, and blue-collar. Employment status was divided into permanent or temporary employment.

#### 2.2.2. Health-Related Characteristics

The following covariates related to physical and psychological health were considered: musculoskeletal pain, fatigue, depression or anxiety, and sleep disturbance. Musculoskeletal pain was recognized when respondents answered ‘yes’ to varying joint or muscle pain. Fatigue was recognized when they answered ‘yes’ to the question of whether they had a headache, eye strain, or general fatigue. Depression or anxiety was recognized if respondents answered ‘yes’ to the question of whether they were depressed or anxious. Sleep disturbance was recognized as the case if they answered ‘Every day’, ‘Many times in a week’, or ‘Many times in a month’ to questions such as ‘Difficult to fall asleep’, ‘Wake up repeatedly while sleeping’, or ‘Wake up with exhaustion or extreme fatigue’ over the last 12 months.

#### 2.2.3. Psychological Well-Being Level

The level of psychological well-being was evaluated through the WHO-5 Well-Being Index. This tool contains five questions that asks about positive feelings during the last two weeks: ‘I have felt cheerful and in good spirits’, ‘I have felt calm and relaxed’, ‘I have felt active and vigorous’, ‘I woke up feeling fresh and rested’, and ‘My daily life has been filled with things that interest me’. Each item was measured on a 6-point Likert scale from ‘At no point in time (0 point)’ to ‘All of the time (5 points)’. This was dichotomized into good and poor well-being and if the total score is 13 points or less, they were classified as ‘poor’ [24].

### 2.3. Data Analysis Method

Data analysis was performed using the software SAS Enterprise Guide 4.3 (SAS Inc., Cary, NC, USA). The psychological well-being level, according to the demographic and health-related characteristics of the subjects was compared by the χ²-test. Multiple logistic regression analysis was conducted to identify factors associated with psychological well-being.

### 2.4. Ethical Consideration

This study was approved by the S University Institutional Review Board (1912-045-01).

## 3. Results

### 3.1. Subject Characteristics

Of the total sample, 60.2% were psychologically healthy (‘good’) and 39.8% were unhealthy (‘poor’). Among the ‘poor’, 2.2% belonged to the 15–29 year old group, 8.1% to the 30–49 year-old group, and 29.5% to the over 50 group. Table 1 shows descriptive statistics for demographic, occupational, and health-related characteristics of participants. In terms of education level, high school graduates were the most common at 35.3%. In the group of 15–29 and 30–49 year olds, the highest proportion was college or more at 6.4% and 16.5%, respectively. On the other hand, a low education level (middle school or less) was the highest (34.1%) in the group of over 50 years old. The most common household type was women living alone (58.5%) and this was the same in all groups. Regarding monthly income, the category 1–1.99 million KRW was the most common (35.5%).

Regarding occupational characteristics, 45.5% were employed in sales and service industries. Sales and service workers were the most common in the 15–29 and 30–49 year-old groups, and blue-collar workers were most likely over 50 years old; 80.3% were temporary employees, 7.1% were shift workers, and 3.1% were night shift employees.

For health-related characteristics, the group over 50 years old had the highest percentages of health-related characteristics such as musculoskeletal pain, fatigue, depression or anxiety, sleep disturbance.

### 3.2. Subject Characteristics and Psychological Well-Being

Table 2 shows differences in subject characteristics and psychological well-being. In the 15–29 year- old group, those with an income of 1–1.99 million KRW or more than 3 million KRW, fatigue, depression or anxiety, and sleep disturbance were more likely to be psychologically unhealthy.

In the 30–49 year-old group, participants with a basic education level (≤high school), living alone or alone with children, with an income of less than 2 million KRW, in a sales and service or blue-collar job, and permanent workers were more likely to be psychologically unhealthy. Regarding health-related characteristics, the psychologically unhealthy group had higher rates of musculoskeletal pain, fatigue, depression or anxiety, and sleep disturbance.

In the over 50 year-old group, participants that had an education of middle school or less, living alone, income of less than 1 million KRW, and blue-collar workers were more likely to be psychologically unhealthy. The percentages of participants who had pain, fatigue, depression or anxiety, and sleep disturbance were significantly higher in the psychologically unhealthy group.

### 3.3. Factors Associated with Psychological Well-Being

Table 3 shows the factors associated with the psychological well-being of female-headed households. In the 15–29 year-old group, the risk of being psychologically unhealthy in case of depression or anxiety was 5.378 times higher (95% CI = 2.223–13.012) than without depression or anxiety. Other variables were not statistically significant.

In the 30–49 year-old group, statistically significant factors included household type, musculoskeletal pain, fatigue, and depression or anxiety. The single head of house living alone had a significantly higher risk of being psychologically unhealthy than those who lived with a spouse and children (OR = 0.495, 95% CI = 0.373–0.657), lived with parents (OR = 0.527, 95% CI = 0.31–0.895), or lived with other family members or relatives (OR = 0.558, 95% CI = 0.364–0.856). Those who had musculoskeletal pain (OR = 1.772, 95% CI = 1.418–2.215), fatigue (OR = 1.551, 95% CI = 1.245–1.932), and depression or anxiety (OR = 2.49, 95% CI = 1.61–3.859) had a higher risk of being psychologically unhealthy than those without these symptoms.

In the over 50 year-old group, statistically significant factors included education level, household type, monthly income, musculoskeletal pain, fatigue, and depression or anxiety. Those with an education of middle school or less had a significantly higher risk of being psychologically unhealthy than high school graduates (OR = 0.7, 95% CI = 0.604–0.81) and college or more (OR = 0.608, 95% CI = 0.477–0.775). There was a higher risk of being psychologically unhealthy among those living alone than those who lived with a spouse (OR = 0.635, CI = 0.516–0.781) or lived with a spouse and children (OR = 0.548, CI = 0.415–0.723). Those who had income of less than 1 million KRW had a higher risk of being psychologically unhealthy than those earning more than 1 million KRW; 1–1.99 million KRW (OR = 0.595, CI = 0.515–0.688), 2–2.99 million KRW (OR = 0.49, CI = 0.404–0.594), or more than 3 million KRW (OR = 0.456, CI = 0.363–0.573). The risks of health-related factors were as high as for being psychologically unhealthy: musculoskeletal pain (OR = 1.571, CI = 1.381–1.787), fatigue (OR = 1.272, CI = 1.121–1.443), or depression or anxiety (OR = 1.919, CI = 1.526–2.414).

## 4. Discussion

Pearce began discussions about the feminization of poverty in the 1970s [25]. According to her report, as of 1975, two out of three poor people in the USA were women, 70% of the elderly poor were women, and more than half of poor households had female heads of house. This is a global trend, and Korea is no exception. In 2018, the rate of female permanent workers (47.4%) were lower than that of male permanent workers (54.3%), and women’s average monthly wage was 68.8% of male wages [5]. Female-headed households were more likely to face a household poverty crisis than male-headed households because they have to manage their livelihoods and bring up their children alone.

Above all, women’s poverty level generally differ from men. In addition to economic factors, non-economic factors contribute to poverty. Therefore, women’s poverty should be discussed in terms of social exclusion rather than from a solely economic perspective. Earlier studies have reported that women’s poverty cannot be identified simply by economic indicators [26]. We should assess the process of poverty, especially in studies on women. Here, the concept of social exclusion extends the definition of traditional poverty, highlighting the existence of social deprivation in the multi-dimensional domains of education, health, environment, housing, and culture, in addition to economic factors [27]. Additionally, women’s poverty is linked to poverty in women’s households, which requires a different understanding than that of poverty in men’s households [4]. Female-headed families are not only likely to experience poverty in terms of economic indicators, but also in non-monetary aspects. They experience overlapping dimensions of poverty, which is understood here as social deprivation. In addition, social deprivation affects the life satisfaction and psychological well-being of individuals.

The WHO-5 well-being index developed by the WHO is regarded as the most appropriate tool for assessing psychological stability and mental health with respect to workers and working environments [28]. According to the study of the psychological well-being of 34 European countries, the following factors were mainly related to poor psychological well-being: quantitative job demands, demands for hiding emotions, low possibilities for development, low meaning of work, low role conflict, low quality of leadership, low social support, low sense of community, job insecurity, low job promotion, work-life imbalance, discrimination, and bullying [29].

This study found that the factors associated with the psychological well-being of Korean female-headed households differed according to age group. In the 15–29 year-old group, the risk factor for psychological well-being was depression or anxiety. In the 30–49 year-old group, statistically significant factors included household type, musculoskeletal pain, fatigue, and depression or anxiety. In the over 50 year-old group, statistically significant factors included education level, household type, monthly income, musculoskeletal pain, fatigue, and depression or anxiety.

In each age group, those who had depression or anxiety had 5.378 times (aged 15–29), 2.493 times (aged 30–49), and 1.919 times (aged over 50) a higher risk of being psychologically unhealthy. In the case of musculoskeletal pain, psychological health was poorer 1.772 times in the 30–49 year-old group, and 1.57 times in the over 50 year-old group. In the case of fatigue, psychological health was poorer, and 1.55 times in the 30–49 year-old group, and 1.27 times in the over 50 year-old group. In particular, psychological well-being was good with higher educational levels and monthly incomes in the over 50 year old group. This confirmed that socioeconomic vulnerability aggravates psychological poor health.

It is noteworthy that as age increased, living alone was a negative factor for psychological health, confirming a different view from earlier studies on family living. For female-headed households, there were many findings on dependents as an economic burden or stressors. It was reported that as the number of children increased, life satisfaction decreased [30] or economic stress reduced quality of life [31]. In the analysis of the National Family Health Survey on female-headed households and poverty reported in the United States, large household size and the child–adult ratio were explained as negatively affecting factors [6]. However, in this study, we found that there were positive impacts on the psychological well-being perceived by female-headed households when they lived with family including a spouse, children, parents, or siblings. This was also confirmed among women aged 30–49 years old, carrying the burden of caring for their children and parents. Through this, it can be inferred that the psychological well-being perceived by female-headed households was not due to simple economic poverty or burdens on family care, but more complex factors. This may be because the characteristics of maternal love positively affected psychological health in the process of raising children [32], and cohabitation with family supported the psychological well-being of the female-headed household [33]. It has been further reconfirmed that family support is a positive and important factor affecting psychological health [34,35]. These findings are in line with the qualitative research [36] showing the problems of female-headed households have become a big threat such as role overload or poverty reproduction as well as an opportunity such as social maturity.

This study showed that the rate of living alone tends to increase with age. The rate of living alone increased from 7.0% among the 15–29 year-old group to 12.3% among the 30–49 year-old group, and to 39.2% among the over 50 year old group. Furthermore, it has been found that the rate of psychological unhealthiness tended to increase.

The increase in women’s life expectancy, together with the aging population, was related to an increase in female-headed households, especially among women living alone. Therefore, psychological health care for women living alone, over age 50 is important. In particular, due to their socio-economic vulnerability, the rate of lower income among them was relatively high at 60.9%, and blue-collar workers were the most common. Regarding health-related factors, they were found to have poor physical health. Earlier studies also pointed out the problems with the well-being of women living alone and reported the need for appropriate interventions [37]. Living with family was a positive factor on the psychological well-being of female heads of house. However, active policy support is also needed such as community resources to help prevent probable health problems in the future. In addition, their health improvement can be achieved through training and helping them to adapt to new and multifaceted roles, providing more economic support and helping them raise their social status [36].

This study analyzed secondary data collected by a self-reported method through a cross sectional survey design. Therefore, it was impossible to assert the causal relationship between variables. In addition, even if results could be predicted through existing statistical methods and earlier studies, there are some limitations: no additional control other than the variables identified through the investigation were implemented. Despite these limitations, it is worth noting that the factors associated with psychological well-being of female -headed households were identified by age group in the process of recognizing the psychological health of female heads of house and trying to support them in multiple dimensions. Based on the results of this study, we need to provide multi-dimensional support at the community and government levels so that female-headed households can lead socially stable lives. In addition, institutional support for female heads of house who live alone, which is a gradually increasing population, should be established.

## 5. Conclusions

The purpose of this study was to identify the psychological well-being of female-headed households by age group and to identify factors that influence them. As a result, factors associated with psychological well-being of female-headed households differed according to age group. As age increased, living alone and physical unhealthiness were negative factors for psychological well-being. It has been found that socio-economic poverty aggravates psychological well-being, especially for women over 50 years old.

The psychological well-being perceived by female households is a complex one that goes beyond economic poverty and burden on dependents. Therefore, a multidimensional support strategy should be included in the concept of social deprivation, and a preventive approach is needed to establish a support system in the community. In addition, as identified in this study, it is necessary to support stability among female-headed households practically through mediation appropriate to each age group’s needs.

## Figures and Tables

**Table 1:** Characteristics of subjects (N = 9084).

Variables	Classification	Total		15–29		30–49		≥50		χ^2^(*p*)
Psychological Well-being	Good	5467	(60.2)	601	(6.6)	1749	(19.3)	3117	(34.3)	282.2718 (<0.0001)
	Poor	3617	(39.8)	197	(2.2)	739	(8.1)	2681	(29.5)	
Education level	Middle school or less	3153	(34.7)	8	(0.1)	52	(0.6)	3093	(34.1)	3576.8259 (<0.0001)
	High school	3208	(35.3)	208	(2.3)	935	(10.3)	2065	(22.7)	
	College or more	2723	(30.0)	582	(6.4)	1501	(16.5)	640	(7.1)	
Household type	Living alone	5314	(58.5)	632	(7.0)	1120	(12.3)	3562	(39.2)	994.3243 (<0.0001)
	Living with spouse	641	(7.1)	12	(0.1)	128	(1.4)	501	(5.5)	
	Living with children	1816	(20.0)	8	(0.1)	586	(6.5)	1222	(13.5)	
	Living with spouse and children	727	(8.0)	4	(0.04)	413	(4.6)	310	(3.4)	
	Living with parents	207	(2.3)	50	(0.6)	95	(1.1)	62	(0.7)	
	Living with others *	379	(4.2)	92	(1.0)	146	(1.6)	141	(1.6)	
Monthly income (million KRW)	<1	2170	(23.9)	82	(0.9)	99	(1.1)	1989	(21.9)	1359.2011 (<0.0001)
	1–1.99	3220	(35.5)	356	(3.9)	780	(8.6)	2084	(22.9)	
	2–2.99	2266	(24.9)	284	(3.1)	934	(10.3)	1048	(11.5)	
	≥3	1428	(15.7)	76	(0.8)	675	(7.4)	677	(7.5)	
Occupation type	Administrator/Expert	562	(6.2)	95	(1.1)	279	(3.1)	188	(2.1)	2185.5136 (<0.0001)
	Office worker	1179	(13.0)	275	(3.0)	666	(7.3)	238	(2.6)	
	Sales & Service worker	4136	(45.5)	373	(4.1)	1269	(14.0)	2494	(27.5)	
	Blue collar job worker	3207	(35.3)	55	(0.6)	274	(3.0)	2878	(31.7)	
Employment status	Permanent	1792	(19.7)	167	(1.8)	310	(3.4)	1315	(14.5)	115.6355 (<0.0001)
	Temporary	7292	(80.3)	631	(7.0)	2178	(24.0)	4483	(49.4)	
Shift work	No	8439	(92.9)	677	(7.5)	2248	(24.8)	5514	(60.7)	145.7147 (<0.0001)
	Yes	645	(7.1)	121	(1.3)	240	(2.6)	284	(3.1)	
Night shift	No	8806	(96.9)	740	(8.2)	2388	(26.3)	5678	(62.5)	74.5193 (<0.0001)
	Yes	278	(3.1)	58	(0.6)	100	(1.1)	120	(1.3)	
Musculoskeletal pain	Not have	5183	(57.1)	669	(7.4)	1839	(20.2)	2675	(29.5)	804.2956 (<0.0001)
	Have	3901	(42.9)	129	(1.4)	649	(7.1)	3123	(34.4)	
Fatigue	Not have	5949	(65.5)	652	(7.2)	1824	(20.1)	3473	(38.2)	240.3417 (<0.0001)
	Have	3135	(34.5)	146	(1.6)	664	(7.3)	2325	(25.6)	
Depression/Anxiety	Not have	8543	(94.0)	770	(8.5)	2388	(26.3)	5385	(59.3)	39.2967 (<0.0001)
	Have	541	(6.0)	28	(0.3)	100	(1.1)	413	(4.6)	
Sleep disturbance	Not have	6615	(72.8)	613	(6.8)	1909	(21.0)	4093	(45.1)	40.1672 (<0.0001)
	Have	2469	(27.2)	185	(2.0)	579	(6.4)	1705	(18.8)	

* Brothers and sisters, relatives, grandparents, grandchildren, friends, etc.; KRW = Korean Won.

**Table 2:** Subject characteristics and psychological well-being.

Variables	Classification	15–29					30–49					≥50				
		Good		Poor		χ^2^(*p*)	Good		Poor		χ^2^(*p*)	Good		Poor		χ^2^(*p*)
		n (%)		n (%)			n (%)		n (%)			n (%)		n (%)		
Education level	Middle school or less	5	(0.8)	3	(1.5)	3.5073 (0.1731)	29	(1.7)	23	(3.1)	24.666 (<0.0001)	1346	(43.2)	1747	(65.2)	291.0363 (<0.0001)
	High school	148	(24.6)	60	(30.5)		612	(35.0)	323	(43.7)		1315	(42.2)	750	(28.0)	
	College or more	448	(74.5)	134	(68.0)		1108	(63.4)	393	(53.2)		456	(14.6)	184	(6.9)	
Household type	Living alone	476	(79.2)	156	(79.2)	0.0706 (0.7905)	762	(43.6)	358	(48.4)	41.4777 (<0.0001)	1754	(56.3)	1808	(67.4)	114.5032 (<0.0001)
	Living with spouse	9	(1.5)	3	(1.5)		96	(5.5)	32	(4.3)		334	(10.7)	167	(6.2)	
	Living with children	2	(0.3)	6	(3.1)		373	(21.3)	213	(28.8)		687	(22.0)	535	(20.0)	
	Living with spouse and children	4	(0.7)	0	(0)		329	(18.8)	84	(11.4)		227	(7.3)	83	(3.1)	
	Living with parents	42	(7.0)	8	(4.1)		75	(4.3)	20	(2.7)		38	(1.2)	24	(0.9)	
	Living with others	68	(11.3)	24	(12.2)		114	(6.5)	32	(4.3)		77	(2.5)	64	(2.4)	
Monthly income (million KRW)	< 1	64	(10.7)	18	(9.1)	8.4063 (0.0383)	60	(3.4)	39	(5.3)	12.305 (0.0064)	778	(25.0)	1211	(45.2)	290.9058 (<0.0001)
	1–1.99	254	(42.3)	102	(51.8)		523	(29.9)	257	(34.8)		1198	(38.4)	886	(33.1)	
	2–2.99	229	(38.1)	55	(27.9)		672	(38.4)	262	(35.5)		682	(21.9)	366	(13.7)	
	≥3	54	(9.0)	22	(11.2)		494	(28.2)	181	(24.5)		459	(14.7)	218	(8.1)	
Occupation type	Administrator/Expert	76	(12.7)	19	(9.6)	1.6677 (0.6441)	208	(11.9)	71	(9.6)	14.3389 (0.0025)	135	(4.3)	53	(2.0)	150.5164 (<0.0001)
	Office worker	202	(33.6)	73	(37.1)		491	(28.1)	175	(23.7)		173	(5.6)	65	(2.4)	
	Sales & Service worker	282	(46.9)	91	(46.2)		878	(50.2)	391	(52.9)		1474	(47.3)	1020	(38.1)	
	Blue collar job worker	41	(6.8)	14	(7.1)		172	(9.8)	102	(13.8)		1335	(42.8)	1543	(57.6)	
Employment status	Permanent	124	(20.6)	43	(21.8)	0.1281 (0.7622)	193	(11.0)	117	(15.8)	10.9613 (0.0011)	676	(21.7)	639	(23.8)	3.7882 (0.055)
	Temporary	477	(79.4)	154	(78.2)		1556	(89.0)	622	(84.2)		2441	(78.3)	2042	(76.2)	
Shift work	No	507	(84.4)	170	(86.3)	0.4319 (0.5679)	1578	(90.2)	670	(90.7)	0.1154 (0.7667)	2953	(94.7)	2561	(95.5)	1.9092 (0.1795)
	Yes	94	(15.6)	27	(13.7)		171	(9.8)	69	(9.3)		164	(5.3)	120	(4.5)	
Night shift	No	554	(92.2)	186	(94.4)	1.1011 (0.3447)	1682	(96.2)	706	(95.5)	0.5425 (0.5027)	3053	(98.0)	2625	(97.9)	0.009 (0.9266)
	Yes	47	(7.8)	11	(5.6)		67	(3.8)	33	(4.5)		64	(2.1)	56	(2.1)	
Musculoskeletal pain	Not have	509	(84.7)	160	(81.2)	1.3212 (0.2652)	1380	(78.9)	459	(62.1)	75.967 (<0.0001)	1749	(56.1)	926	(34.5)	269.9036 (<0.0001)
	Have	92	(15.3)	37	(18.8)		369	(21.1)	280	(37.9)		1368	(43.9)	1755	(65.5)	
Fatigue	Not have	505	(84.0)	147	(74.6)	8.7837 (0.0041)	1364	(78.0)	460	(62.3)	65.7907 (<0.0001)	2079	(66.7)	1394	(52.0)	129.7207 (<0.0001)
	Have	96	(16.0)	50	(25.4)		385	(22.0)	279	(37.8)		1038	(33.3)	1287	(48.0)	
Depression/Anxiety	Not have	592	(98.5)	178	(90.4)	29.0876 (<0.0001)	1708	(97.7)	680	(92.0)	42.8294 (<0.0001)	2981	(95.6)	2404	(89.7)	77.6157 (<0.0001)
	Have	9	(1.5)	19	(9.6)		41	(2.3)	59	(8.0)		136	(4.4)	277	(10.3)	
Sleep disturbance	Not have	473	(78.7)	140	(71.1)	4.8581 (0.0322)	1371	(78.4)	538	(72.8)	9.0801 (0.0031)	2270	(72.8)	1823	(68.0)	16.1933 (<0.0001)
	Have	128	(21.3)	57	(28.9)		378	(21.6)	201	(27.2)		847	(27.2)	858	(32.0)	

KRW = Korean Won.

**Table 3:** Factors associated with psychological well-being.

Variables	Classification	OR(CI)					
		15–29		30–49		≥50	
Education level	Middle school or less	1		1		1	
	High school	0.583	(0.128–2.654)	0.837	(0.458–1.53)	0.7	(0.604–0.81) *
	College or more	0.408	(0.092–1.813)	0.716	(0.387–1.323)	0.608	(0.477–0.775) *
Household type	Living alone	1		1		1	
	Living with spouse	0.752	(0.175–3.229)	0.734	(0.477–1.13)	0.635	(0.516–0.781) *
	Living with children	4.073	(0.704–23.572)	1.068	(0.854–1.337)	1.145	(0.989–1.325)
	Living with spouse and children	<0.001	(<0.001->999.999)	0.495	(0.373–0.657) *	0.548	(0.415–0.723) *
	Living with parents	0.59	(0.265–1.312)	0.527	(0.31–0.895) *	0.938	(0.548–1.606)
	Living with others	1.019	(0.597–1.741)	0.558	(0.364–0.856) *	0.947	(0.663–1.354)
Monthly income (million KRW)	<1	1		1		1	
	1–1.99	1.45	(0.734–2.864)	0.856	(0.536–1.366)	0.595	(0.515–0.688) *
	2–2.99	0.925	(0.439–1.952)	0.75	(0.463–1.216)	0.49	(0.404–0.594) *
	≥3	1.667	(0.698–3.979)	0.739	(0.449–1.218)	0.456	(0.363–0.573) *
Occupation type	Administrator/Expert	1		1		1	
	Office worker	1.261	(0.698–2.278)	1.069	(0.766–1.493)	0.909	(0.584–1.416)
	Sales & Service worker	0.96	(0.522–1.767)	1.097	(0.796–1.513)	1.234	(0.862–1.766)
	Blue collar job worker	1.081	(0.463–2.524)	1.257	(0.834–1.895)	1.134	(0.781–1.647)
Employment status	Permanent	1		1		1	
	Temporary	0.83	(0.502–1.372)	0.816	(0.615–1.083)	1.143	(0.997–1.312)
Shift work	No	1		1		1	
	Yes	0.949	(0.501–1.795)	0.675	(0.447–1.019)	0.924	(0.662–1.29)
Night shift	No	1		1		1	
	Yes	0.761	(0.313–1.855)	1.363	(0.75–2.476)	1.38	(0.842–2.262)
Musculoskeletal pain	Not have	1		1		1	
	Have	0.945	(0.571–1.565)	1.772	(1.418–2.215)*	1.571	(1.381–1.787) *
Fatigue	Not have	1		1		1	
	Have	1.533	(0.962–2.444)	1.551	(1.245–1.932) *	1.272	(1.121–1.443) *
Depression/Anxiety	Not have	1		1		1	
	Have	5.378	(2.223–13.012) *	2.493	(1.61–3.859) *	1.919	(1.526–2.414) *
Sleep disturbance	Not have	1		1		1	
	Have	1.431	(0.964–2.126)	1.152	(0.934–1.422)	1.097	(0.971–1.24)

KRW = Korean Won; * *p < 0.05*.
